# Inhibitory effects of *Bacillus velezensis* ID-A01 supernatant against *Streptococcus mutans*

**DOI:** 10.1186/s12866-023-03114-2

**Published:** 2023-11-24

**Authors:** Hyeoungeun Kim, Chi-Young Han, Su-Hyeon Eun, Min-Goo Kim, A-Rang Im, Byeonghun Lee

**Affiliations:** 1 Research Laboratories, Ildong Pharmaceutical Co., Ltd, 20, Samsung 1-ro 1-gil, Hwaseong-si 18449, Hwaseong-si, Gyeonggi-do Republic of Korea; 2 Department of Chemical and Biomolecular Engineering, Sogang University, 35 Baekbum-ro, Mapo-gu, Seoul 04107, Republic of Korea

**Keywords:** *Bacillus velezensis*, Antimicrobial, Biofilm, *Streptococcus mutans*, Dental caries, Oral health

## Abstract

**Background:**

Dental caries is a chronic oral disease caused by microbial infections, which result in erosion of the dental enamel and cause irreversible damage. Therefore, proper disease management techniques and the creation of an environment that prevents intraoral growth and biofilm formation of *Streptococcus mutans* in the early stages, are crucial to prevent the potential progression of dental plaque to disease. Here, we aimed to investigate antimicrobial and antibiofilm effects of the *Bacillus velezensis* ID-A01 supernatant (ID23029) against *S. mutans*, and its inhibitory effects on acidogenesis.

**Results:**

A killing kinetics assay showed a peak lethality percentage of 94.5% after 6 h of exposure to ID23029. In sucrose-exposed conditions, ID23029 inhibited lactic acid formation, preventing the pH from falling below the threshold for enamel demineralization, and inhibited up to 96.6% of biofilm formation. This effect was maintained in the presence of lysozyme. Furthermore, ID23029 retained up to 92% lethality, even at an intraoral concentration at which lysozyme is ineffective against *S. mutans*.

**Conclusions:**

This study demonstrates the potential of the *B. velezensis* ID-A01 supernatant for the prevention and treatment of dental caries. Its eventual use in dental practice is encouraged, although further studies are required to confirm its beneficial effects.

## Background

Dental caries is a bacterial infectious disease wherein intraoral bacteria feed on food scraps that remain in the mouth after brushing the teeth and break them down to generate acid, causing tooth erosion and decay. Considering the high global morbidity of the disease, with approximately 60% of adults [1], 50% of students [2], and almost 100% of 6–7-year-old children [3] affected, preventive measures are highly warranted.

*Streptococcus mutans* is one of the primary species of bacteria that cause dental caries [4, 5]. *S. mutans* produces glucan, the main component of biofilms, from glucose through a glucose metabolism process mediated by glycosyltransferases (GTFs), such as GtfB, GtfC, and GtfD. *S. mutans* then combines with other microbes involved in biofilm formation (e.g., *S. gordonii* and *Actinomyces* spp [6]., facilitating the secondary attachment by acid-producing intraoral commensal bacteria (e.g., *S. sanguinis* and *S. salivarius*) [7]. In addition, *S. mutans* attaches to the enamel on the tooth surface, inducing the formation of microbial communities [8], and expresses virulence genes, thus negatively affecting oral health.

*Bacillus spp.* are Gram-positive spore-forming bacteria that, through genome mining, generate various secondary metabolites, such as polyketide synthase, non-ribosomal peptide, and lipopeptides. These substances are used in various fields for their antimicrobial, anti-inflammatory, and anticancer properties [9]. In particular, the antimicrobial effects of *Bacillus spp.* are effective against various diseases, including intraoral inflammatory disease [10].

Once the tooth surface becomes coated by substances such as glycoproteins, acidic proline-rich proteins, mucins, bacterial cell debris, exoproducts, and sialic acid, some bacteria (e.g., *S. sanguis* and *A. viscosus*) colonize the tooth surface through cell-to-surface interactions. *S. mutans* forms biofilms by inducing cell-to-cell interactions within the colony [11]. During this process, *S. mutans* breaks down sucrose to glucose and fructose, which are then converted to lactic acid via the phosphotransferase system [12]. *S. mutans* excretes lactic acid extracellularly to protect its own cells from damage. Lactic acid then triggers a neutralizing reaction with tooth enamel, which is primarily hydroxyapatite (Ca_10_(PO_4_)_6_(OH)_2_), dissolving calcium and phosphorus and forming dental caries.

Herein, we aimed to investigate the antimicrobial activity of the *B. velezensis* ID-A01 supernatant (ID23029) against *S. mutans* and its ability to inhibit formation of biofilms, which is a primary cause of dental caries. We also aimed to investigate the mechanism underlying the inhibition of lactic acid production and whether this effect was maintained in the presence of lysozyme, in order to evaluate the potential of ID23029 for controlling *S. mutans* biofilms and preventing the development of dental caries.

## Results

### Bacterial susceptibility assay

**Fig. 1:**
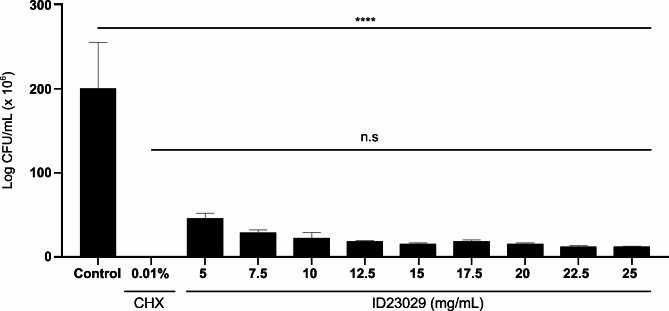
Bacterial susceptibility of ID23029 against *Streptococcus mutans*. Results are expressed as the mean ± SD of triplicate samples. ****p < 0.0001 compared with the control group. n.s. compared with the CHX group

Figure 1 shows growth of *S. mutans* at different ID23029 concentrations. Compared with the viable count of the untreated *S. mutans* (control), the lethality percentage in the 0.01% CHX conditions was around 100%. Moreover, when *S. mutans* was treated with ID23029, the cell lethality percentage was 94% at a concentration of 25 mg/mL and remained above 90% on all 10% serial dilutions even at a concentration of 12.5 mg/mL.

### Time-kill assay

**Fig. 2:**
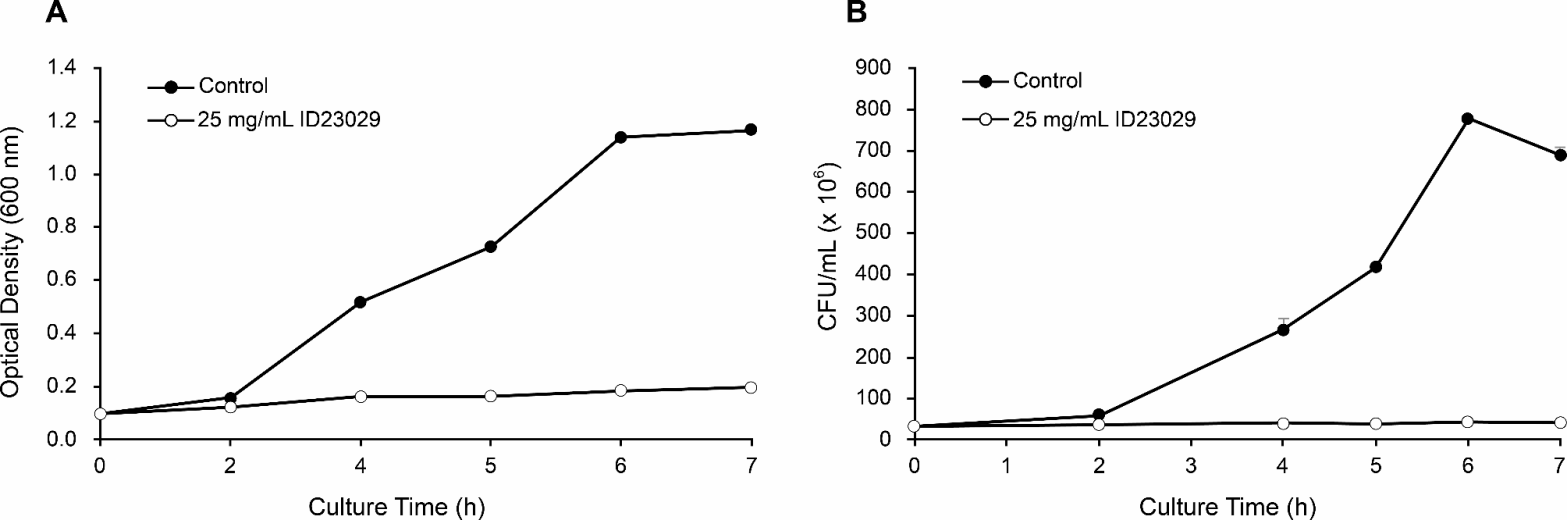
Bacterial time-kill assay results for *Streptococcus mutans* treated with ID23029. The plots are based on the absorbance at 600 nm **(a)** and on the surviving curve of planktonic *S. mutans***(b)**

Figure 2 shows the results of the killing kinetics analysis of ID23029 against *S. mutans*. When OD_600nm_ absorbance and viable count were examined over time (Fig. 2a and b), *S. mutans* showed a lag growth phase within the first 2 h after inoculation, an exponential growth period between 2 and 6 h, and a stationary phase after 6 h. Conversely, when treated with ID23029, *S. mutans* failed to enter the exponential phase and did not grow.

### Lactic acid concentration test

**Fig. 3:**
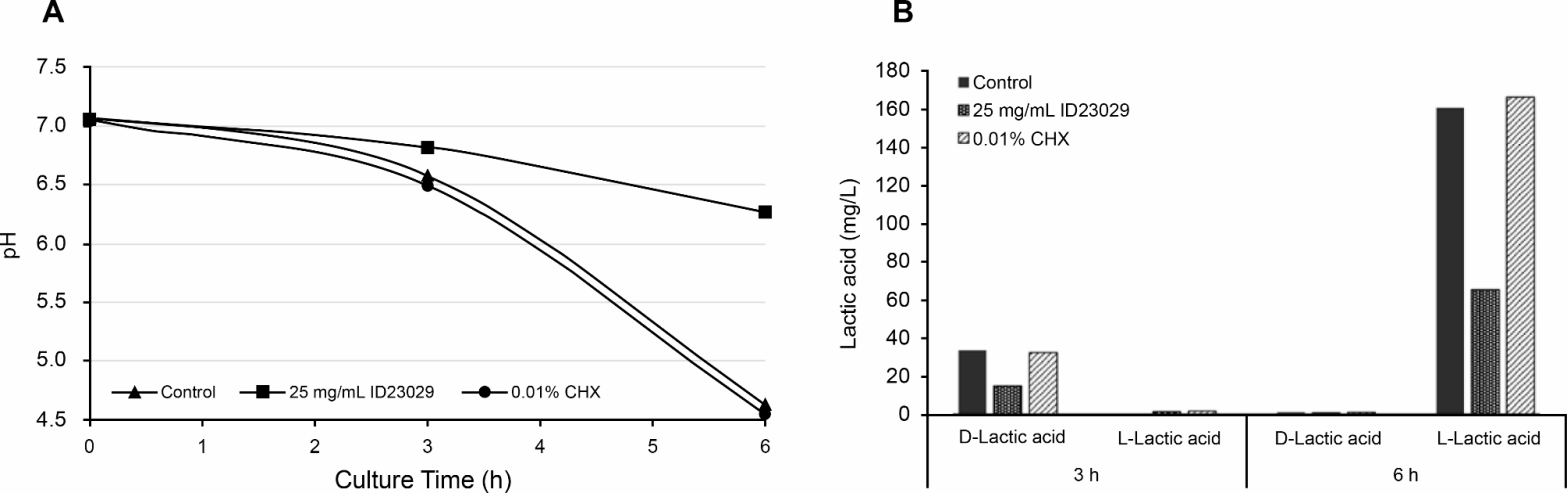
Effect of ID23029 on *Streptococcus mutans* acid production. pH decline over time **(a)**. Analysis of the D- and L-lactic acid content changes according to the incubation time **(b)**

We used a glycolytic pH drop assay and analyzed the lactic acid content to investigate the changes in the *S. mutans* metabolite levels induced by the ID23029 treatment (Fig. 3). The pH of the culture with 1% sucrose was acidic (pH 4.0) after 6 h (T_6_) compared with that at baseline (T_0_, pH 7.1); however, the pH of the group treated with ID23029 was neutral (pH 6.3) at T_6_. *S. mutans* produces D-/L-form lactic acid as it grows; ID23029 treatment inhibited the expression of D- and L-lactic acids by approximately 56% and 59%, respectively. This finding demonstrates that ID23029 can inhibit lactic acid expression and protect against the negative effect of the reduced pH level, which is a major cause of enamel demineralization.

### Antibiofilm assay

**Fig. 4:**
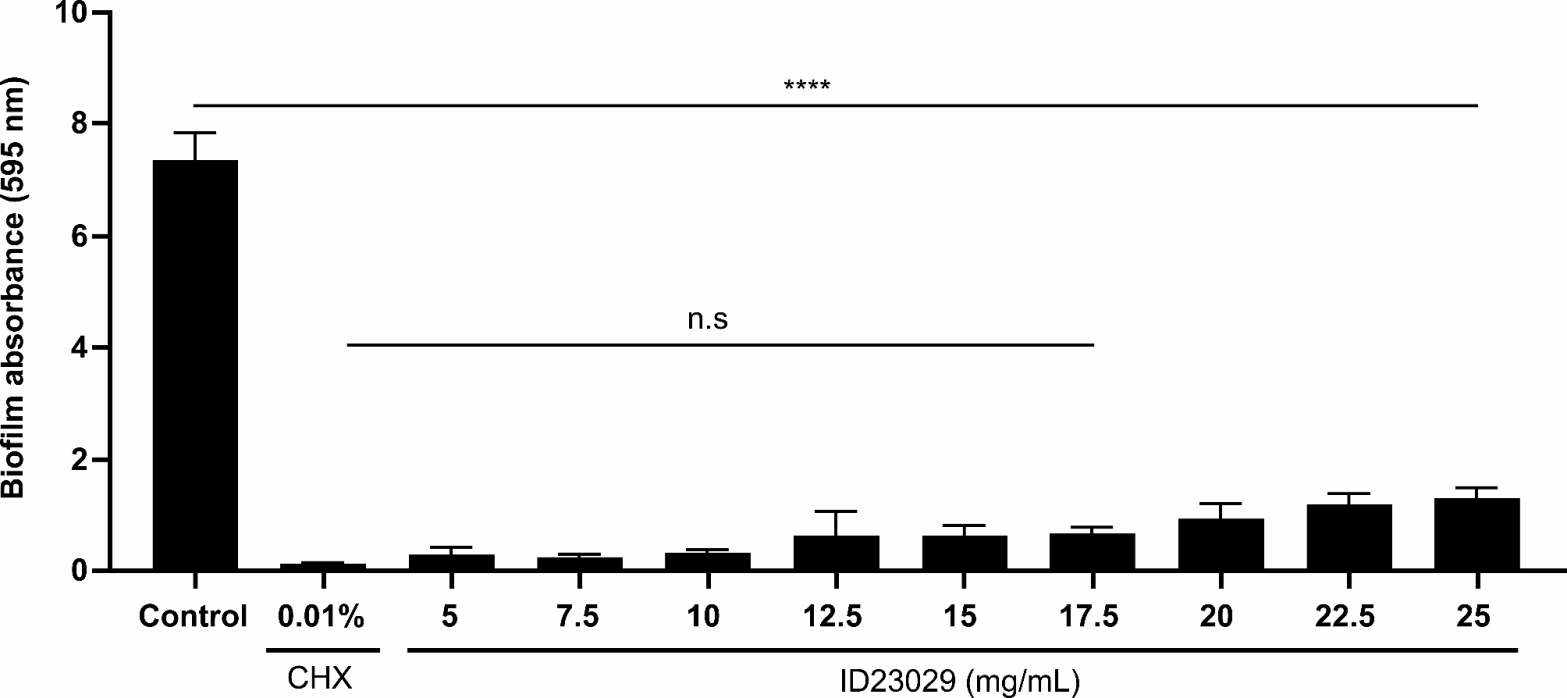
*Streptococcus mutans* -induced biofilm assay in the presence of sucrose. The *S. mutans* biofilm formation was measured. Significant differences from the control (untreated) are indicated with ****p < 0.0001. n.s compared with the CHX group

We analyzed the inhibition of biofilm formation induced by different ID23029 concentrations (Fig. 4). The absorbance of biofilms increases in proportion to the matrix volume. The 7.5 mg/mL ID23029 group showed the lowest absorbance, demonstrating a 97% inhibition rate compared with controls. In the concentration interval of 12.5–25 mg/mL, the rate of inhibition increased further with each dilution, and the peak inhibition rate in this interval was 91.5%.

### Confocal laser scanning microscopy and scanning electron microscopy observation of the biofilm

**Fig. 5:**
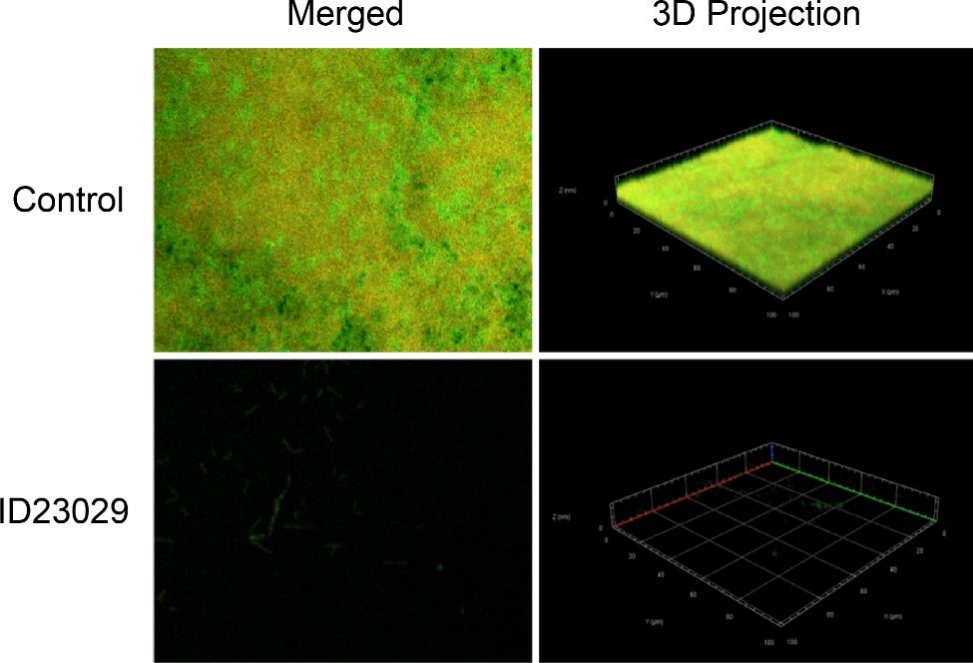
Confocal laser scanning microscopy and live/dead staining results. The confocal laser scanning microscopy micrographs illustrate the effect of ID23029 on the biofilm architecture and the bacterial cell viability, which can be assessed based on the fluorescence (live and dead bacteria are stained green and red, respectively). The biofilms were incubated for 72 h after treatment with 25 mg/mL of ID23029

**Fig. 6:**
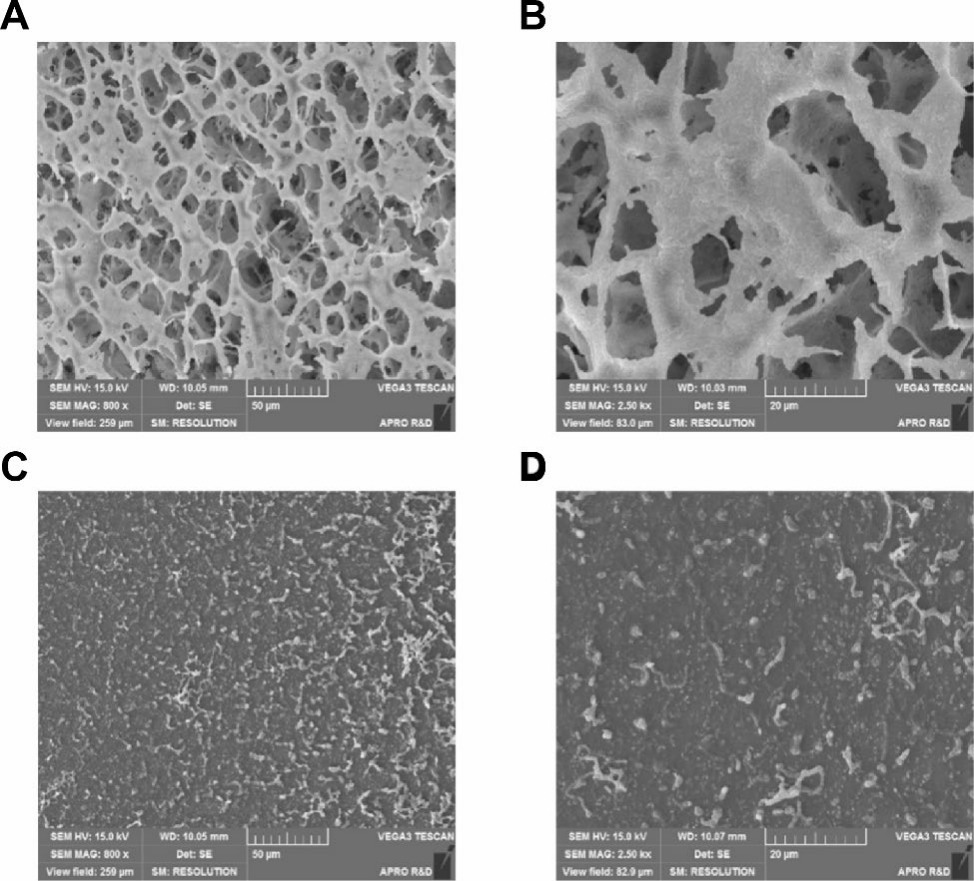
SEM morphological observations of the *Streptococcus mutans* biofilms. The effect of ID23029 on biofilm formation and architecture. The untreated control biofilms were confirmed by the images at ×800 magnification **(a)** and 2,500× **(b)**. The biofilms of the test group were incubated for 72 h after treatment with 25 mg/mL of ID23029, and their formation was confirmed by the images at a magnification of 800× **(c)** and 2,500× **(d)**

We used confocal laser scanning microscopy (CLSM) and scanning electron microscopy (SEM) to investigate differences in the appearance of *S. mutans* biofilms treated with ID23029 (Figs. 5 and 6). Both analyses demonstrated that ID23029 has antibiofilm effects. Under controlled conditions, live and dead bacteria aggregated to form a 3D net-like biofilm matrix. Conversely, the ID23029 treatment reduced the number of live bacteria, preventing the formation of a 3D structure.

### Lysozyme tolerance test

**Fig. 7:**
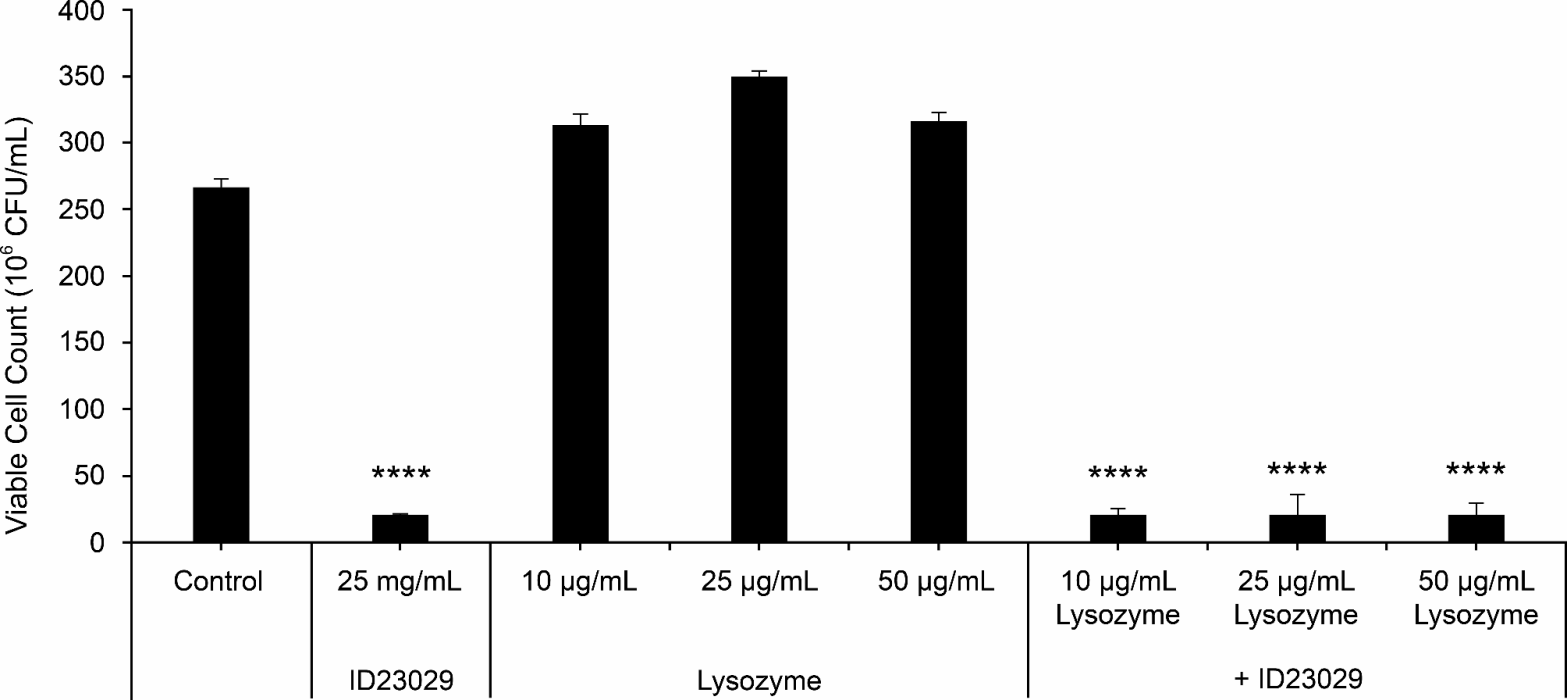
Growth effect of *Streptococcus mutans* on ID23029 exposed to varying concentrations of lysozyme. The results are expressed as the mean ± SD of triplicate samples. ****p < 0.0001 compared with the control group

To investigate the stability of ID23029 in the presence of lysozyme, ID23029 was reacted with 10–50 µg/mL of lysozyme for 16 h at 37℃. The resulting mixture was used to treat the *S. mutans*, and the viable count was analyzed (Fig. 7). Compared with the growth of untreated *S. mutans*, the group treated with ID23029 alone showed a 92.2% growth-impairment rate. In the group exposed to 50 µg/mL lysozyme, the growth-impairment rate was 91.9%, suggesting that ID23029 treatment remained effective even in this concentration value.

## Discussion

Oral microbiome studies have identified 500–700 species of microbes that live in the oral cavity [13]. When the normal microbiome becomes unbalanced, an environment beneficial for the growth of pathogenic bacteria is likely to be formed, which ultimately leads to oral diseases [14]. Dental caries, which is one of the main oral diseases alongside periodontitis, refers to the enamel damage caused by the acid produced as a metabolite by microbes attached to the tooth surface. The World Health Organization emphasized that dental caries affects almost 60–90% of students and most adults globally and is a major contributor to the loss of natural teeth in older adults [15].

In some clinical trials and animal experiments, fluorine compounds, such as chlorhexidine and triclosan, were reported to affect the bacterial membranes and enzymes and to inhibit biofilm formation by impairing bacterial metabolism [16]. Although chemical treatment methods with broad-spectrum antimicrobial effects are often selected for periodontal disease, these can be toxic and cause adverse effects with long-term use. Indeed, excessive exposure to fluoride can lead to dental and skeleton damage and fluorosis [16]. There has emerged a growing interest in the use of natural products for oral disease to address these issues, and several studies on this subject have been published [17].

Here, we investigated the antibacterial, antibiofilm, and acid-reducing effects of the *B. velezensis* ID-A01 supernatant against *S. mutans* (KCTC 3065). First, we investigated the antimicrobial effects of ID23029 against platonic *S. mutans* and observed a peak lethality of 96%, which remained above 90% even after a 50% dilution (12.5 mg/mL; Fig. 1). Notably, at all treatment concentrations of ID23029, we found no significant difference in efficacy compared to chlorhexidine, which is one of the most commonly used substances to treat tooth decay [18, 19]. When we examined the growth curves over time, *S. mutans* showed a lag phase during the first 2 h, followed by an exponential growth phase between 2 and 6 h (Fig. 2). Moreover, *S. mutans* treated with ID23029 did not show an exponential growth phase, and the viable count and absorbance remained stagnant. Therefore, ID23029 inhibited the growth and division of *S. mutans*, preventing the viable count from increasing and thus demonstrating antimicrobial activity.

The acidogenicity of *S. mutans* is a physiological outcome of its glucose metabolism and induces the demineralization of the tooth surface. When the accumulation of acid decreases the pH of the local intraoral environment to below the 5.0–5.5 threshold, it causes tooth demineralization and decay [20]. Here, ID23029 delayed the decrease in pH, and the final pH remained above the aforementioned threshold. Moreover, when we investigated the lactic acid content, the D- and L-lactic acid levels demonstrated reduction rates of 56% and 59%, respectively (Fig. 3).

Biofilm is one of the main pathogenic characteristics of *S. mutans* and is an important cause of dental caries [5]. Biofilms are composed of a complex, multidimensional structure of glucans and fructans synthesized during the glucose metabolism of *S. mutans* [21]. This multidimensional glucan structure makes the biofilm thicker and firmer and plays a decisive role in plaque maturation [22]. *S. mutans* forms a firm biofilm when cultured on a polystyrene surface for 72 h with sucrose (data not shown); we then investigated the effects of ID23029 on a biofilm in identical conditions. The antibiofilm effect of ID23029 differed depending on its concentration, with a peak efficacy of 96.6% being observed at a concentration of 7.5 mg/mL (Fig. 4). Interestingly, the results differed from those of antimicrobial activity, which also showed a dose-dependent correlation. In the 12.5–25 mg/mL concentration range, the effectiveness of ID23029 in preventing biofilm formation increased further with each dilution. This indicates that some insoluble materials, which differ from the substances responsible for the antimicrobial effects of ID23029, are major biofilm formation-suppressing factors. Since the adhesivity of *S. mutans* occurs owing to “pathogenicity determinants,” protein expression in biofilm-forming strains of the bacteria differs from that in planktonic *S. mutans* [3]. The insoluble component of ID23029 was assumed to act on adhesion-expressing *S. mutans* and its expressed proteins. Next, we used CLSM to verify whether ID23029 had both antimicrobial and antibiofilm effects (Fig. 5). In the *S. mutans* control, live and dead bacteria aggregated to form a 3D biofilm structure; after treatment with ID23029, the overall number of live bacteria decreased, and the bacteria were unable to form a 3D biofilm. The appearance of the biofilm matrix could easily be observed using SEM (Fig. 6). These findings indicate that ID23029 has antimicrobial and antibiofilm effects.

Lysozyme is a muramidase that acts on the peptidoglycan wall of Gram-positive bacteria to induce cell death. It is present in high quantities in tears and saliva, playing a partial role in inducing innate immunity. The mean concentration of lysozyme in saliva is 28 µg/mL, irrespective of age or sex [23]. Consequently, the intraoral concentration of lysozyme does not provide primary defense against the adhesion of *S. mutans*; however, ID23029 is not degraded by enzymes and maintains its efficacy against *S. mutans* in their presence (Fig. 7). This study clearly demonstrates that ID23029 has antimicrobial and antibiofilm activity and inhibits acid production against *S. mutans*, a caries-causing pathogen that is much more potent than other oral bacterial species. These results strongly support the hypothesis that ID23029 could help prevent tooth decay.

## Conclusion

ID23029, a *B. velezensis* ID-A01 supernatant, exhibits antimicrobial, antibiofilm, and anti-acid production effects against *S. mutans*. In addition, it does not cause adverse effects typically induced by oral disinfectants and chemical treatments and has stable effects against intraoral enzymes such as lysozyme. Therefore, ID23029 can be potentially used for the long-term prevention and treatment of dental caries. In future studies, the effects of ID23029 on the inhibition of glycosyltransferase gene expression, which is a mechanism of biofilm formation associated with glucose metabolism, should be investigated.

## Methods

### Bacterial strains and growth conditions

*B. velezensis* ID-A01 (NCBI accession number CP066377.1) was isolated from kimchi, a traditional Korean fermented food. *B. velezensis* ID-A01 was successively cultured in tryptic soy broth (TSB; MB cell, Korea). *B. velezensis* ID-A01 was inoculated at 1% (v/v) with TSB and cultured at 37℃ with continuous shaking for 42 h under aerobic conditions.

The *S. mutans* KCTC 3065 strain was obtained from the Korean Collection for Type Cultures (KCTC), inoculated on brain heart infusion (BHI) broth (MBcell, MB-B1008) at 10% (v/v), incubated in 5% CO_2_, incubator at 37℃ for 16 h.

### Preparation of the cell-free culture supernatant lyophilisate ID23029

After cultivation, the cells were harvested via centrifugation (32,300 × *g*, 10 min, 4℃), and the supernatant pH was adjusted to pH 6.5 using 75% phosphoric acid. The supernatant was filtered using a 0.2-µm filter (Sartorius, Germany) to remove cell debris.

The cell-free culture supernatant was subjected to overnight freezing at − 80℃ and was freeze-dried for 88 h using a freeze-dryer (Operon Alliance Instruments, Korea). ID23029 (25 mg/mL in distilled water) was finally obtained and stored in a 4℃ cooler until further use.

### Bacterial susceptibility assay

ID23029 concentrations were continuously diluted with 10% sterile distilled water to evaluate the growth inhibitory activity for *S. mutans*. These serial dilutions were then treated with an *S. mutans* mixture at a concentration of 10% (v/v) that had been inoculated on fresh BHI broth. The viable count was measured after 24 h of culture. *S. mutans* (KCTC 3065) used in this experiment was inoculated on BHI at 10% (v/v) and incubated in 5% CO_2_ conditions at 37℃ for 16 h before use. The negative control group was treated with water, and the positive control was chlorhexidine (Sigma Aldrich, 282,227) diluted to 0.01%. The experiment was conducted in 5-mL round tubes using a volume of 3 mL. To analyze the viable count, the mixture was diluted in saline (3 M, WH40005786), poured in BHI agar plates, and cultured at 37℃ for 48 h, before counting the number of colonies formed. The results were expressed as *S. mutans* colony forming units (CFU) for each ID23029 concentration. All conditions were tested in triplicate.

### Time-kill assay

The killing kinetics of ID23029 were investigated using a partially modified time-kill assay [24]. The *S. mutans* (KCTC 3065) was inoculated on BHI at 10% (v/v) and incubated at 37℃ with 5% CO_2_ for 16 h. The suspension was then diluted to 6 × 10^7^ CFU/mL, followed by treatment with 25 mg/mL of ID23029. The OD_600nm_ and viable count were analyzed over time.

### Lactic acid concentration test

To investigate the ability to reduce the production of acidic compounds, which are a major cause of dental caries, we performed a glycolytic pH drop assay and measured the lactic acid concentration. After culturing the *S. mutans* (KCTC 3065) overnight on BHI, the culture medium was then inoculated at 1% (v/v) on BHI broth treated with 1% sucrose, and the supernatant was collected over time. The pH and lactic acid content were analyzed at different culture times (0, 3, and 6 h). The lactic acid content was measured using a D/L-lactic acid (D-/L-lactate) assay kit (Megazyme, K-DLATE), in accordance with the manufacturer’s instructions. The test, negative control, and positive control groups were treated with 25 mg/mL of ID23029, water, and 0.01% (v/v) chlorhexidine (Sigma, 282,227), respectively. All treatments were applied at a concentration of 10% (v/v). The experiment was conducted in 15-mL conical tubes using a volume of 8 mL.

### Antibiofilm assay

To assess for biofilm formation inhibition, a crystal violet (CV) assay was performed. After overnight culture on BHI, an *S. mutans* (KCTC 3065) suspension at an OD_600nm_ of 1.28 (6 × 10^8^ CFU/mL) was inoculated at a concentration of 1% (v/v) on BHI broth containing 1% sucrose. A 96-well plate was prepared by treating ID23029 diluted in series from 2.5 mg/mL; the total volume reached 150 µL/well on adding the *S. mutans* mixture. Water and 0.01% chlorhexidine were added to the negative and positive control groups, respectively. The plates were cultured at 37℃ and incubated in 5% CO_2_; the medium was replaced with fresh 1% sucrose BHI broth every 24 h to ensure a continuous sucrose supply.

After 72 h, the plates were washed twice with PBS and fixed with methyl alcohol for 30 min. After washing them again with PBS, the plates were treated with 0.1% CV (Sigma, 548-62-9) containing 20% ethyl alcohol for 20 min, washed twice with tap water, and dissolved in ethyl alcohol, before measuring the OD_595nm_ to measure the biomass. Each condition was replicated thrice, and the results were expressed as the mean absorbance.

### Confocal laser microscopic imaging

To investigate the efficacy of ID23029 against biofilm formation and *S. mutans* (KCTC 3065) in biofilms, CLSM was used. After treating a 6 × 10^6^ CFU/mL *S. mutans* suspension with ID23029 and inoculating it on BHI broth containing 1% sucrose, the culture was incubated at 37℃ with 5% CO_2_ for 72 h to induce biofilm formation. Thereafter, live and dead bacteria were stained with SYTO® 9 green-fluorescent and red-fluorescent propidium iodide. The samples were imaged using CLSM at magnification 63x in a dark room, and three-dimensional (3D) images were produced using Z-stacks (ZEN 2.6, Blue edition).

### Scanning electron microscopic imaging

To investigate the effects of ID23029 on the *S. mutans* biofilm structure, SEM was used. To facilitate metal coating, which is an essential step prior to the SEM scan, we placed a cover slip on the plate wells used for the experiment and induced biofilm formation on the cover slip. Thereafter, the cover slip was washed, moved to a new plate, and freeze-dried before imaging.

### Lysozyme tolerance test

To test the stability of ID23029 against lysozyme, which is a hydrolytic enzyme that defends the body against infection, a tolerance test was performed. We incubated 10–50 µg/mL of lysozyme (human, Sigma, L1667), ID23029, and a ID23029 + lysozyme mixture at 37℃ for 16 h. Then, we added these substances, at 10% (v/v) dilution, to fresh BHI broth containing 6 × 10^8^ CFU/mL of *S. mutans* (KCTC 3065) that had been cultured overnight. We incubated the cultures for 24 h and measured the viable count in triplicate. The experiment was performed in 5-mL round tubes at a volume of 3 mL.

### Statistical analysis

At least three independent experiments were performed. Statistical analysis was performed through one-way analysis of variance using GraphPad Prism 8 software. All data were presented as the mean ± standard deviation, and a p value of < 0.05 was considered significant.

## Data Availability

The datasets used and/or analyzed during the current study are available from the corresponding author on reasonable request.

## Abbreviations

BHI: brain heart infusion
CLSM: confocal laser scanning microscopy
CV: crystal violet
GTFs: glycosyltransferases
KCTC: Korean Collection for Type Cultures
SEM: scanning electron microscopy
